# Investigating spatio-temporal mobility patterns and changes in metro usage under the impact of COVID-19 using Taipei Metro smart card data

**DOI:** 10.1007/s12469-021-00280-2

**Published:** 2021-08-16

**Authors:** Christian Martin Mützel, Joachim Scheiner

**Affiliations:** 1 Faculty of Spatial Planning, Technische Universität Dortmund, 44227 Dortmund, Germany; 2 Department of Transport Planning, Faculty of Spatial Planning, Technische Universität Dortmund, 44227 Dortmund, Germany

**Keywords:** Taiwan, Public transport, Smart card, Spatio-temporal flow maps, COVID-19, Mobility

## Abstract

Modern public transit systems are often run with automated fare collection (AFC) systems in combination with smart cards. These systems passively collect massive amounts of detailed spatio-temporal trip data, thus opening up new possibilities for public transit planning and management as well as providing new insights for urban planners. We use smart card trip data from Taipei, Taiwan, to perform an in-depth analysis of spatio-temporal station-to-station metro trip patterns for a whole week divided into several time slices. Based on simple linear regression and line graphs, days of the week and times of the day with similar temporal passenger flow patterns are identified. We visualize magnitudes of passenger flow based on actual geography. By comparing flows for January to March 2019 and for January to March 2020, we look at changes in metro trips under the impact of the coronavirus pandemic (COVID-19) that caused a state of emergency around the globe in 2020. Our results show that metro usage under the impact of COVID-19 has not declined uniformly, but instead is both spatially and temporally highly heterogeneous.

## Introduction

Metro systems are among the most important transportation systems around the world. In big cities thousands or even millions of trips are conducted by metro every day. Compared to other public transit modes, metro systems are characterized by higher speeds and larger capacities, thus attracting high passenger demand (Sun et al. 2015a). Therefore, metro systems have a major influence on urban mobility and are an integral part of life in big cities. Understanding demand patterns is crucial for transport planners and public transit operators to determine an appropriate supply of public transit and enhance reliability. Also, gaining deeper knowledge about urban mobility patterns can help urban planners to build more attractive and sustainable cities.

Understanding urban mobility requires detailed trip data. Traditional public transit data collection methods suffer from high costs, incomplete, inaccurate and inconsistent data (Fonzone et al. 2016). However, modern public transit systems are often run with automated fare collection (AFC) systems in combination with smart cards. As a by-product, these systems passively collect a massive amount of detailed spatio-temporal trip data which can increase our knowledge about mobility patterns, thus opening up new possibilities for public transit planning and management and for urban planners (El Mahrsi et al. 2017; Li et al. 2015; Luo et al. 2017; Pelletier et al. 2011; Sobral et al. 2019; Yu et al. 2019).

The aim of this study is to perform an in-depth investigation of spatio-temporal station-to-station metro trip patterns taking actual geography into account and clearly indicating magnitudes of passenger flow. Our work is based on a case study for Taipei Metro in Taipei City and New Taipei City, Taiwan, using trip data from the AFC smart card system EasyCard. Our analysis covers a week as a whole, divided into several time slices. Based on simple linear regression and line graphs, days of the week and times of the day with similar temporal passenger flow patterns were identified. By comparing flows for January–March 2019, which represent normal conditions, and for January–March 2020, we look at changes in metro trips under the impact of the first wave of the coronavirus pandemic (COVID-19). To the best of our knowledge, this study is the first to comprehensively map spatio-temporal metro trip patterns and spatio-temporal changes in metro usage at this level of detail, and among the first to do so under the impact of COVID-19.

**Fig. 1:**
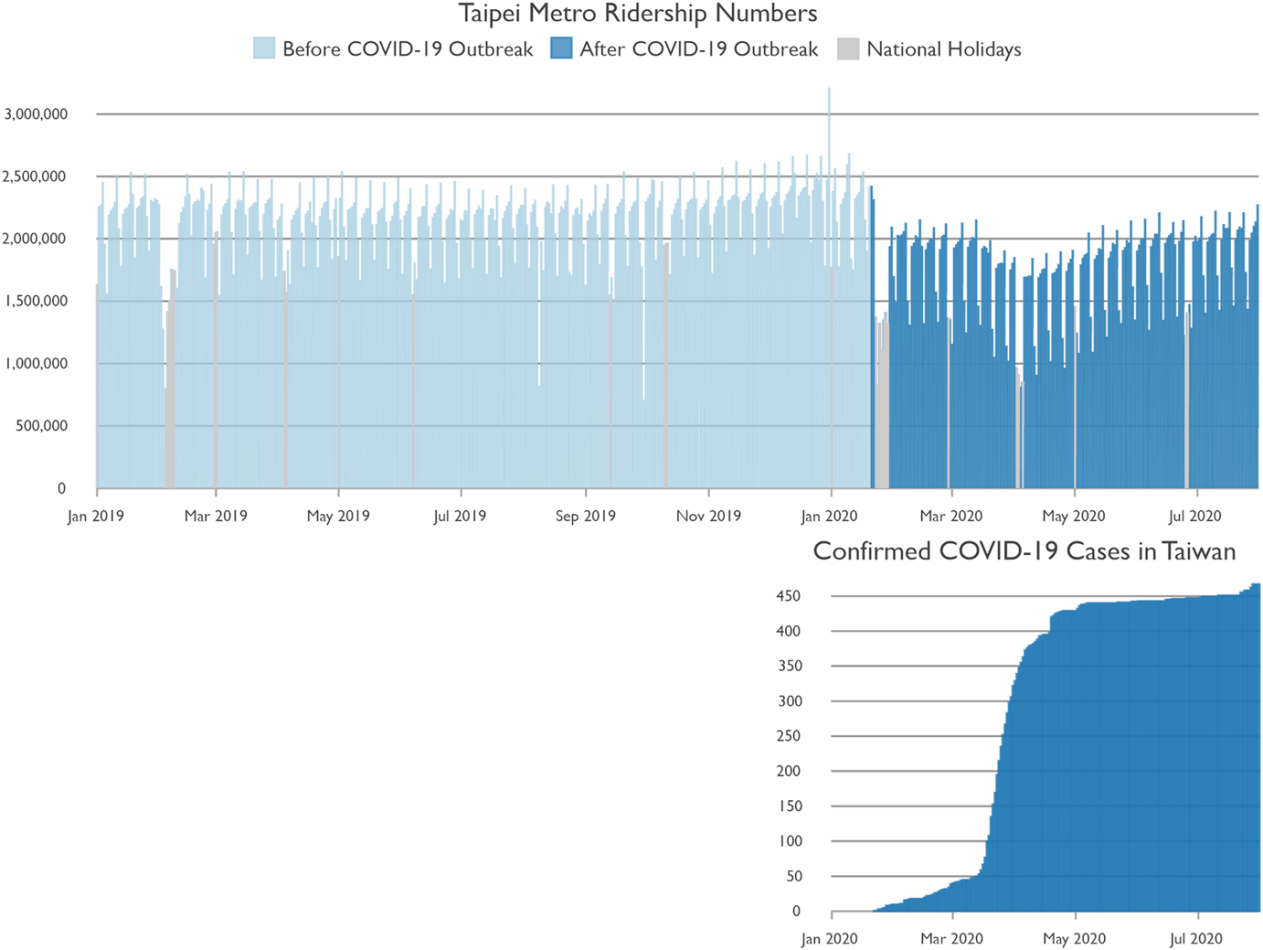
Comparison of the number of confirmed COVID-19 cases in Taiwan and Taipei Metro daily passenger volume. Data Source: Johns Hopkins University (2021), Taipei Rapid Transit Corporation (2020b)

COVID-19 has caused a state of emergency around the globe. At the end of December 2019 the World Health Organization picked up information about a ‘viral pneumonia of unknown cause’ in China (World Health Organization 2020). This disease, which was later named COVID-19, has since developed into a pandemic with more than 163 m confirmed cases and has caused more than 3.38 m. deaths worldwide as of 17 May 2021 (Johns Hopkins University 2021; World Health Organization 2020). The first confirmed COVID-19 case in Taiwan was registered on January 21st 2020, and thus Taiwan was one of the first countries outside of China reached by the disease (Timeline: COVID-19 in Taiwan 2020; Johns Hopkins University 2021). Yet, due to quick reactions and the implementation of strict measures (Timeline: COVID-19 in Taiwan 2020), Taiwan was able to handle the pandemic very well. This makes the case of Taiwan unique, as Taiwan has an extremely low number of confirmed COVID-19 cases and has basically controlled COVID-19 within its national borders. Despite the success of the fight against COVID-19 in Taiwan, the pandemic has left its mark on public transit usage. As public transit often involves crowded and enclosed spaces with limited ventilation, users could be exposed to higher risks of catching COVID-19, although the limited available evidence to date suggests that the use of public transit does not appear to result in an increased risk of infection, at least under the current circumstances of low ridership levels and increased physical distance in vehicles and stations (Charité Research Organisation 2021; Institut Pasteur 2021). Ridership has plummeted around the world since the outbreak of COVID-19 (Beck and Hensher 2020; Follmer et al. 2020; Molloy et al. 2021; Transit 2020). Despite the extremely low number of confirmed COVID-19 cases in Taiwan, Taipei Metro ridership numbers dropped as soon as COVID-19 reached Taiwan (Fig. 1). Many people seem to have immediately reacted to the news of the COVID-19 outbreak and use the metro less than before. Even though the COVID-19 outbreak in Taiwan could be very quickly controlled, metro ridership numbers have not recovered and remain at a significantly lower level. This shows that COVID-19 could have long-term effects on public transit usage.

The remainder of this paper is structured as follows. Section 2 reviews studies conducted on metro smart card trip data. Section 3 introduces Taipei Metro as the case study area, the data used, and the applied methodology. The results are presented in Section 4. Finally, Section 5 summarizes results and discusses policy conclusions.

## Related studies

As a comprehensive representation of public transit usage, data collected by AFC smart card systems has attracted much attention in research especially in the last decade (see Sun et al. 2016, for a useful overview). Two distinct, related topics are the investigation of factors influencing transit usage by means of regression analysis (Chen et al. 2019a; Choi et al. 2012; Jun et al. 2015; Kim et al. 2018; Lin and Shin 2008; Singhal et al. 2014; Zhao et al. 2014a) and passenger forecasting (Li et al. 2018; Liu et al. 2018; Liu et al. 2019; Liu and Chen 2017; Sun et al. 2015b; Wei and Chen 2012). Furthermore, metro smart card data has been used to estimate origin-destination matrices for metro AFC systems where passengers tap their smart cards only when boarding but not when alighting the metro train (Cheng et al. 2020). Metro smart card trip data has also been applied to validate travel demand models (Cho et al. 2015), and to measure public transit service quality in terms of excess journey time (Zhao et al. 2013).

Metro smart card trip data has been combined with socio-economic data gained from surveys to predict the demographic attributes of smart card users (Zhang and Cheng 2018), to infer the employment status of passengers (Zhang and Cheng 2020), to analyze the associations between demographic attributes and travel patterns (Goulet-Langlois et al. 2016), and to identify commuters among passengers of a metro line (Mei et al. 2020). Other studies have used metro smart card trip data to predict lifestyles of passengers based on trip patterns (Shin 2020), to identify home and/or work locations of passengers (Li et al. 2015; Sari Aslam et al. 2019; Zou et al. 2018), to assess the spatial distribution of commuting trips and jobs-housing ratios (Zheng et al. 2019), and to infer trip purpose and activity (Lee and Hickman 2014; Zou et al. 2018).

Another wide research field is the spatio-temporal analysis of metro smart card trip data. Several studies have investigated travel regularity or travel variability of metro users (Cui and Long 2019; Ma et al. 2013; Manley et al. 2018; Zhao et al. 2014a, b, 2017; Zhong et al. 2015). Some authors have applied cluster analysis to investigate the spatio-temporal travel characteristics of passengers (Chen et al. 2019b; Yang et al. 2019), or to identify communities/metro stations based on the spatio-temporal travel patterns (El Mahrsi et al. 2017; Kim et al. 2014; Kim 2020; Zhong et al. 2014, 2015). While cluster analysis allows a reduction in complexity and facilitates making sense of smart card trip data, some information about metro mobility is lost due to the clustering processes. In contrast, we make a visualization approach with our study in order to reveal information which might be lost in a cluster analysis.

Many studies have applied visual analysis techniques to explore the spatio-temporal patterns of metro trips. However, existing data visualization approaches have five major drawbacks:

1. Only a subset of metro trips, such as a subset of origin-destination links (Chen et al. 2019b; Yang et al. 2019) or transit users (Long et al. 2016) is analyzed and visualized.
2. The analysis does not cover specific time slices over a time period with a typical periodicity such as a whole week (Chen et al. 2019b; El Mahrsi et al. 2017; Gong et al. 2012; Huang et al. 2017; Kim et al. 2014; Kim 2020; Long et al. 2016; Ma et al. 2017; Peng and Zou 2020; Yang et al. 2019; Yu et al. 2019).
3. Metro mobility is analyzed at station level, but station-to-station (or area-to-area) links are not investigated (El Mahrsi et al. 2017; Gong et al. 2012; Ma et al. 2017; Peng and Zou 2020; Yu et al. 2019; Zhong et al. 2014).
4. No clear indication of the magnitude of passenger flow is provided for station-to-station (or area-to-area) interactions (Chen et al. 2019b; Huang et al. 2017; Kim et al. 2014; Kim, 2020; Long et al. 2016; Sun et al. 2016; Yang et al. 2019; Zhong et al. 2014).
5. The visualization approach chosen either does not take actual geography into account (Sun et al. 2016; Zhong et al. 2014), or is based on a schematic network diagram (Huang et al. 2017; Sun et al. 2016; Yu et al. 2019), so that no or only rudimentary observations about spatial implications and spatial interactions can be made.

We address these gaps by analyzing network-wide spatio-temporal metro usage at a station-to-station level for a whole week divided into several time slices while taking actual geography into account and clearly indicating magnitudes of passenger flow. Maps, as powerful tools for visual analysis, can help to understand large and complex spatial datasets, discover patterns and outliers, and recognize flows in space and time (Andrienko et al. 2010; Keim et al. 2010; Sun et al. 2016; Robinson et al. 2017; Yu et al. 2019). We create a series of radial flow maps, an exploratory analysis technique (Guo 2009) that shows origins and destinations linked by a line indicative of a route to visualize the quantity of movement between places (Dent et al. 2009; Field 2018).

## Materials and methods

### Study area

**Fig. 2:**
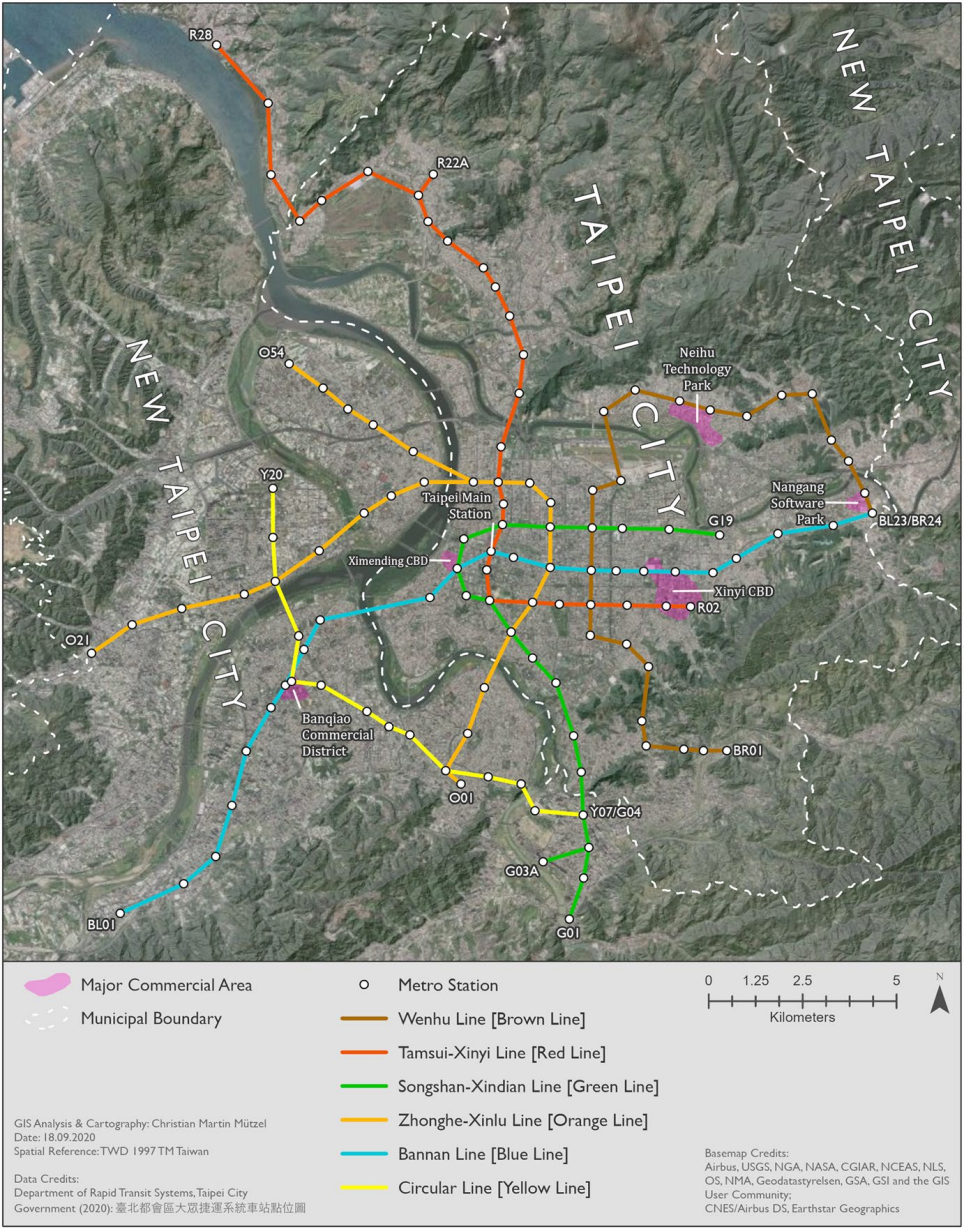
Study area Taipei Metro (metro network simplified to straight-line connections)

The case study was conducted for Taipei Metro, which serves Taipei City and urban parts of New Taipei City as shown in Fig. 2. Taipei City is the capital city and economic, political and cultural center of Taiwan, located in the north of Taiwan main island and completely surrounded by New Taipei City. Both cities are special municipalities in the administrative structure of Taiwan. Despite being two different entities with separate administration systems, Taipei City and New Taipei City are undergoing a regional integration process. Together, they form a twin-city connected by transportation networks (Li et al. 2016).

Taipei City has 2.6 m. inhabitants living in an area of 272 km^2^, resulting in a very high population density of 9732 people/km^2^ (Department of Budget, Accounting and Statistics, Taipei City Government 2020). New Taipei City has roughly 4 m. inhabitants. However, since New Taipei City encompasses a large area of 2053 km^2^, its population density of 1947 people/km^2^ is comparatively low (Department of Budget, Accounting and Statistics, New Taipei City Government 2019). While most of New Taipei City’s urban areas actually are very densely populated, vast parts are mountainous areas with only smaller settlements. The historical city center of Taipei City is near Taipei Main Station. Today, Taipei City’s central business districts are Ximending and Xinyi. Two other major commercial areas in Taipei City are Neihu Technology Park and Nangang Software Park (Li et al. 2016). A band running from Ximending CBD eastwards to Xinyi CBD can best be described as the center of Taipei City today. The Banqiao Commercial District is the center of New Taipei City.

There are three metro systems serving Taipei City and New Taipei City: Taipei Metro, New Taipei Metro, and Taoyuan Airport MRT. The data used in this study only covers Taipei Metro, and thus New Taipei Metro (1 line: Danhai LRT, opened in 2018) and Taoyuan Airport MRT (1 line, opened in 2017) are not considered. Taipei Metro, which has been operating since 1996 and has since been gradually extended, encompasses an operating network length of 146.2 km with 6 lines (Taipei Rapid Transit Corporation 2020d, e). Each metro station has its unique code consisting of an abbreviation indicating the color name of the metro line and a station number. If transfer stations are only counted once, then Taipei Metro network has a total of 119 stations (including 16 transfer stations) in 2020 and had 108 stations (including 13 transfer stations) in 2019. The newest extension of the Taipei Metro network is the first part of the Circular Line [Yellow Line], which had its inauguration on January 31st 2020 (Taipei Rapid Transit Corporation 2020e). Other than that, no changes in metro service were observed during the period investigated. In order to promote the new metro line, passengers enjoyed free rides on the Circular Line [Yellow Line] from January 31st 2020 to February 29th 2020 (Chen and Chiang 2020). It is remarkable that there are no parallel links in the Taipei metro network, meaning that every network link is only served by one line. Also, all transfer stations are served by exactly two lines and no more, including BL12/R10 Taipei Main Station. In 2016 Taipei Metro had a modal share of 19.4% of all trips in Taipei City and 13.6% of all trips in New Taipei City (Ministry of Transportation and Communications, Republic of China (Taiwan) 2016), thus accounting for a relatively large share of all trips.

### Data and data preprocessing

This study uses origin-destination passenger count data by Taipei Rapid Transit Corporation (2020c) which is based on the AFC smart card system EasyCard. The data was acquired for January–March 2019, which represents normal conditions, and for January to March 2020, which is when COVID-19 reached Taiwan. The origin-destination passenger count matrices include the date, the station entry hour, the boarding station, the alighting station, and the respective passenger count. The time of day in the dataset includes 21 station entry hours which cover 05:00 am to 01:00 am of the following day.

Taipei Metro only starts operating at 06:00 am, and only very few stations are still served at 01:00 am (Taipei Rapid Transit Corporation 2020a). Therefore, the origin-destination passenger count matrices station entry hour was manipulated as follows: records of 05:00 am were manipulated to 06:00 am, since these most likely are passengers who enter the metro station early to take the first service of the day, and records of 01:00 am were manipulated to 00:00 am in order to prevent distortions when calculating average passenger volumes per hour because only very few stations are still served after 01:00 am. Records where the boarding and alighting station are identical were removed.

Furthermore, a Taipei Metro stations shapefile (spatial reference: TWD 1997 TM Taiwan) by the Department of Rapid Transit Systems, Taipei City Government (2020) which contains the name of each station and its point location was acquired. The shapefile did not include BL01 Dingpu station, which was manually digitalized. Moreover, some transfer stations were included twice (once for each metro line). In this case the transfer stations were edited to appear as one station, since the origin-destination passenger count matrices do not differentiate this detail.

Both the origin-destination passenger count matrices and the metro stations data were translated from Chinese into English, following the English names in the official Taipei Metro route map. All data used in this study is freely available as open data.^1^

A subset of the origin-destination passenger count data was created by first removing the weeks before the first COVID-19 case was confirmed in Taiwan on January 21st 2020 (Timeline: COVID-19 in Taiwan 2020), and starting the subset with a Monday. The last date of the subset was selected to be the last Sunday of March, thus ensuring that complete weeks are covered. The 2019 subset was created accordingly. All weeks containing national holidays (Directorate-General of Personnel Administration, Executive Yuan 2018, 2019) were separately excluded for 2019 and 2020. The 2019 period from January 28th to February 3rd does not include a national holiday but it was still excluded because the following national holiday falls on a Monday, meaning that the weekend of February 2nd and 3rd could also be considered part of the national holiday. This left a time period covering 7 complete weeks for 2019 and for 2020, respectively. The final subset comprises a total of 26,340,342 records representing 192,858,481 metro trips.

It is unfortunate for the analysis that the outbreak of COVID-19 in Taiwan fell exactly at the time of the Lunar New Year, the biggest festivity in Chinese culture and the longest national holiday in Taiwan. Although this means that there is not a single day of January left in the 2020 data subset, it was still decided to include January in the 2019 subset to ensure that a 7-week period is considered in both years. Another coincidence is that the opening of the new Circular Line [Yellow Line] falls in the study period. The effects of COVID-19 and the effects of this metro network extension can, at least locally, not be clearly separated from each other. However, previous research has shown that the opening of new metro lines tends to increase metro ridership numbers (Fu and Gu 2018; Gao et al. 2019), which means that it can be assumed that if the new Circular Line [Yellow Line] had not opened at the time, passenger volumes would have decreased even more strongly.

### Methods

As metro usage is characterized by weekly periodicity (Fig. 1), the 7-week passenger counts of 2019 and 2020 were averaged by day of the week, station entry hour, boarding station and alighting station to smooth extreme values caused by one-time events.

Analyzing spatio-temporal patterns of metro usage for 7 days of the week and 19 station entry hours per day results in 133 time slices to be mapped for each year of observation, and thus is cumbersome. Therefore, the data needs to be aggregated. Gong et al. (2012) introduced the Hillinger coefficient to measure the similarity of passenger flows between different days. However, even between days with distinctly different temporal passenger flow profiles the Hillinger coefficients are extremely similar, making interpretation difficult. Kim et al. (2018) and Zhong et al. (2015) applied Pearson correlation for this purpose. This also results in high correlations even between days with different temporal passenger flow profiles. Thus, we applied a similar approach by using simple linear regression, combined with line graphs.

We used the R^2^ values to investigate how well the temporal passenger flow profile of one day predicts that of another day. However, since the R^2^ values are based only on variations in profiles between days, but do not take into account similarity in the total passenger counts, we additionally investigated line graphs for metro ridership at the level of the total network on a visual basis. Relying on line graphs only is insufficient as this does not allow similarity to be quantified.

According to this step each origin-destination passenger count was assigned to a day-of-the-week group and time-of-the-day group (see Sect. 4.1). Finally, the average passenger volume per hour and the percentage change in passenger volume under the impact of COVID-19 within each group were calculated per origin-destination link.

## Results

### Network-wide temporal passenger flow patterns

**Fig. 3:**
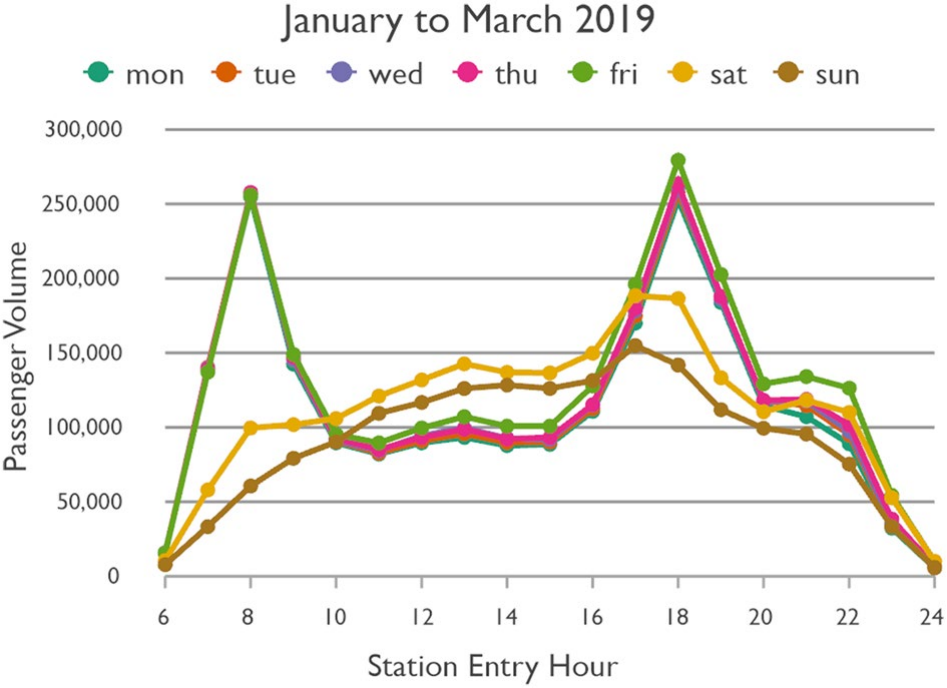
Taipei Metro ridership in a normal condition

**Fig. 4:**
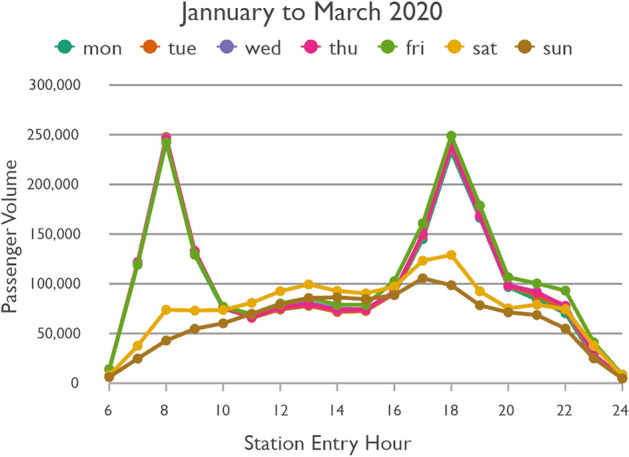
Taipei Metro ridership under the impact of COVID-19

**Fig. 5:**
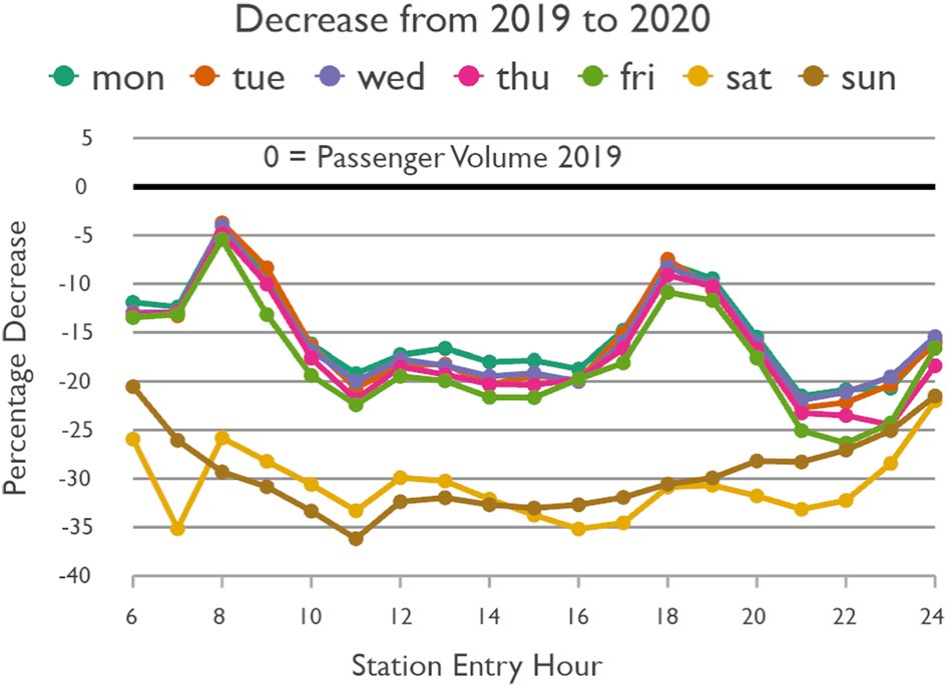
Decrease in Taipei Metro ridership under the impact of COVID-19

Figure 3 shows the temporal passenger flow profiles of every day of the week in a normal condition. The weekdays follow very much the same patterns. They are characterized by two typical peaks representing the morning and evening rush hours. The evening rush hour has slightly higher passenger volumes than the morning rush hour. After the morning rush hour, Friday has slightly higher passenger volumes than the other weekdays, most pronounced at night. The weekend profiles are distinctly different. Saturday and Sunday have relatively similar patterns, but total passenger volume on Saturday is higher than on Sunday and the curve on Sunday is smoother. Figure 4 shows the profiles under the impact of COVID-19. While the total passenger volume dropped, the daytime variations followed those under normal conditions. The percentage decline in passenger volume is shown in Fig. 5. On weekdays the rush hours were clearly affected the least, whereas passenger volume at night dropped the most. On weekends the passenger volume plummeted. Again, whereas the curve of Sunday is relatively smooth, the curve of the decline in passengers on Saturday is characterized by several abrupt changes.

Trips during rush hours typically represent mandatory trips to/from work, school, etc. Still, considering that public transit is most crowded during weekday rush hours and thus the chance of catching COVID-19 could be relatively high, one might expect that passengers would have switched to other modes of transport, such as car or scooter (if available), walking or private bicycle, or Taiwan’s popular bikesharing system YouBike. But this seems to rarely have been the case, as the drops in counts are minor, thus indicating that people either lacked other feasible transportation options, or that they simply accepted the risk.

**Fig. 6:**
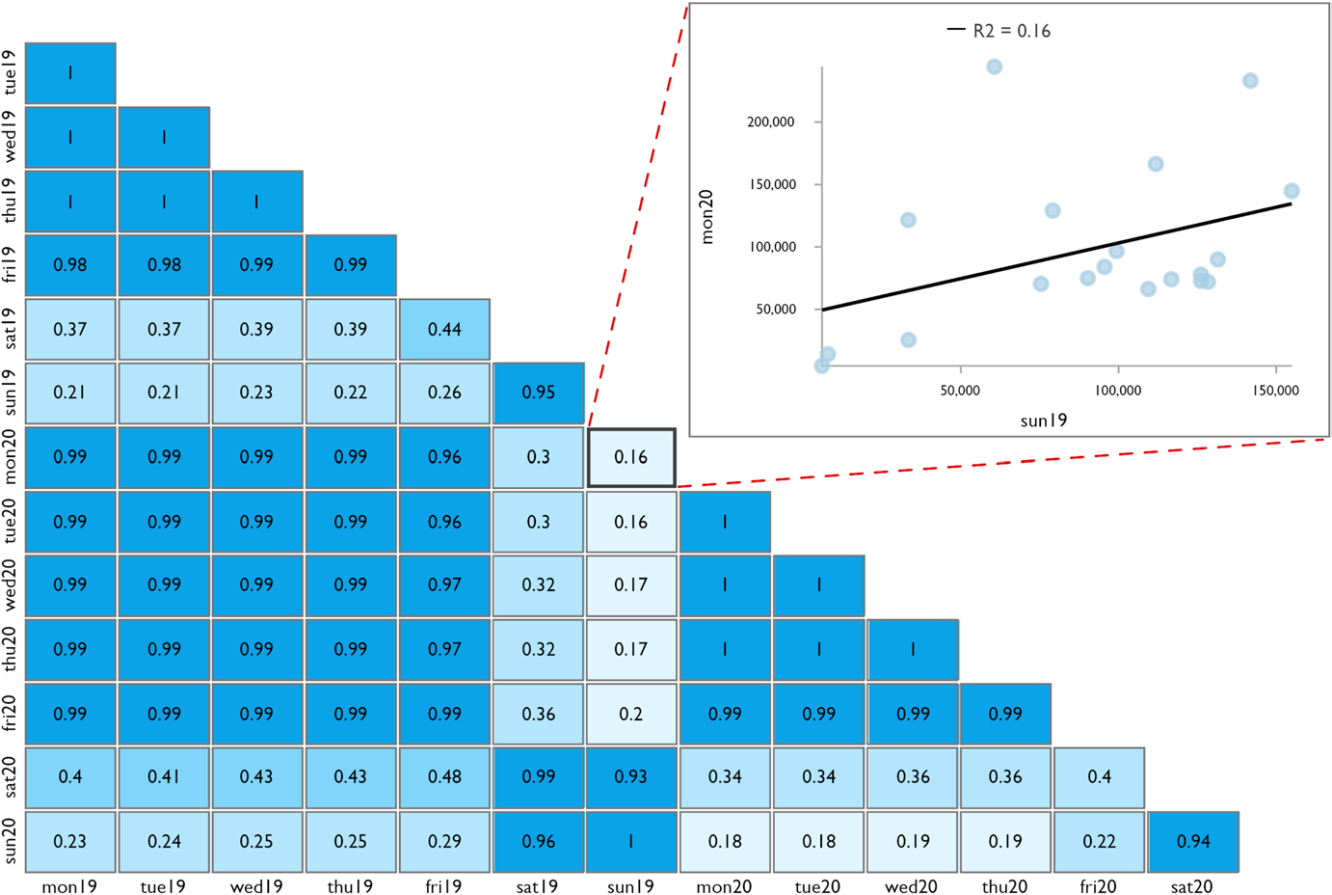
R^2^ between the Taipei Metro temporal passenger flow profiles in the course of different days

Another indicator of the similarity of temporal passenger flows between days is the R^2^ of the simple linear regression as shown in Fig. 6. The flow profiles of Monday to Friday can predict each other extremely well (R^2^ ≥ 0.96). The weekend stands out as distinctly different (R^2^ ≤ 0.48). Saturday and Sunday can predict each other very well (R^2^ ≥ 0.93), although not quite as well as the weekdays. The observations are true for all cases, compared within the year 2019, within the year 2020 and between the years 2019 and 2020.

The results indicate that Monday to Friday could be grouped together, as Fridays differ only to a minor degree from other weekdays. However, we analyze Saturday and Sunday separately, as they have distinct characteristics (see Figs. 3, 4 and  5). Based on the pronounced changes of passenger flow in the line graphs, time-of-the-day groups were identified as shown in Table 1 based on station entry time. Sunday has no clearly pronounced changes in passenger flow throughout the day. For ease of comparability we decided to follow the same groups as for the other days.

**Table 1:** Grouping days of the weeks and times of the day with similar temporal passenger flow patterns

Weekday	Station entry hour	Comment
Monday to Friday	06:00 to 09:59 10:00 to 15:59	Morning rush hour
	16:00 to 19:59 20:00 to 00:59	Evening rush hour
Saturday	06:00 to 09:59 10:00 to 15:59 16:00 to 19:59 20:00 to 00:59	Weak morning rush hour
Sunday	06:00 to 09:59 10:00 to 15:59 16:00 to 19:59 20:00 to 00:59	

### Station-to-station spatio-temporal passenger flow patterns

Using the time slices identified in Sect. 4.1, we conducted an in-depth spatio-temporal analysis of metro trip patterns by creating a series of radial flow maps.

#### Metro usage under normal conditions

**Fig. 7:**
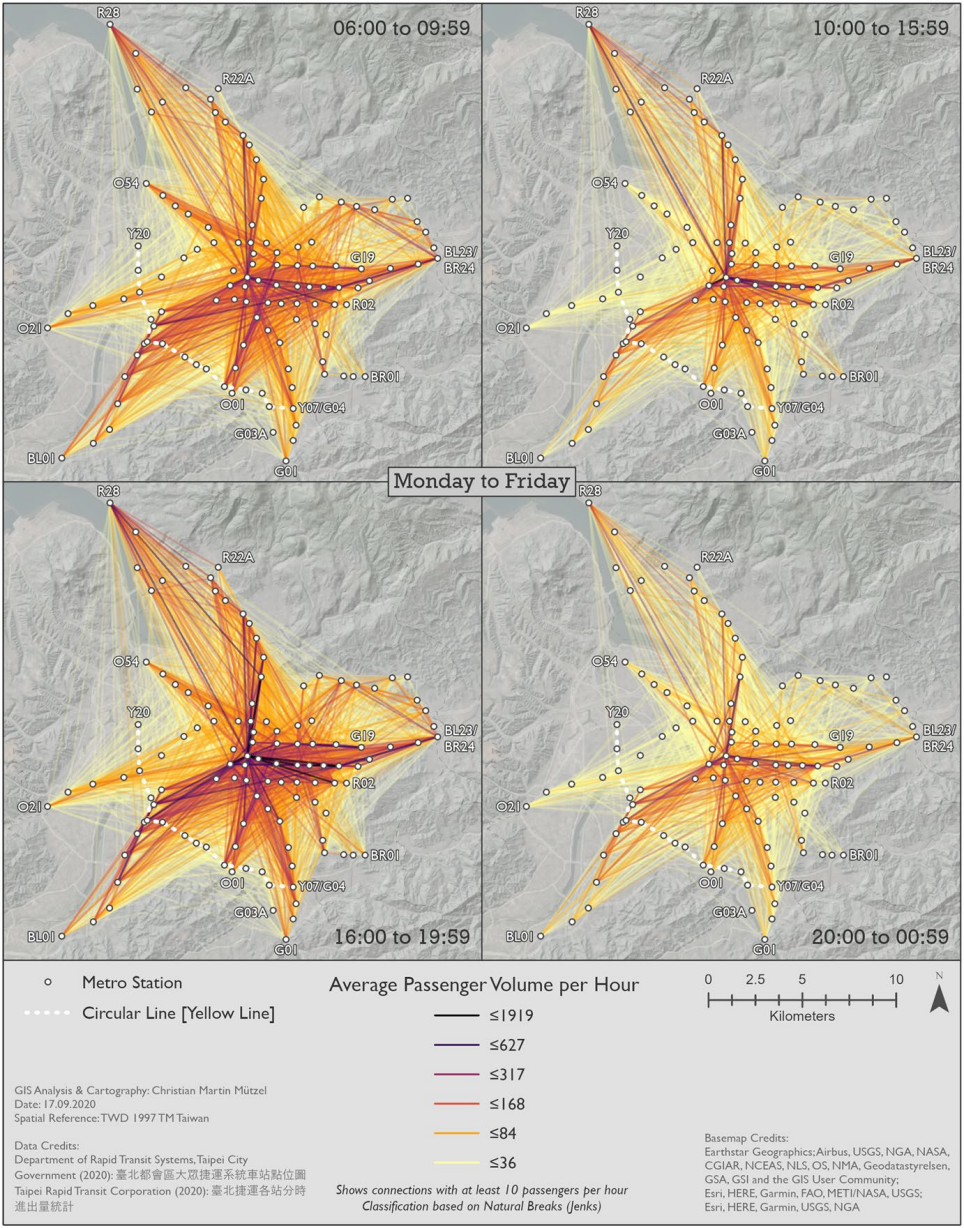
Station-to-station passenger flow in normal conditions on Monday–Friday

**Fig. 8:**
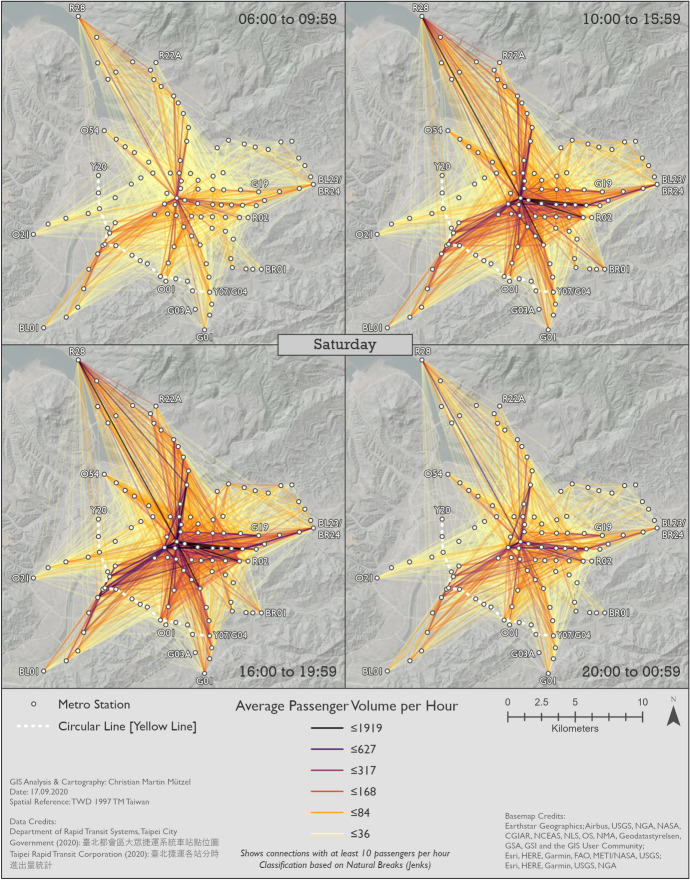
Station-to-station passenger flow in normal conditions on Saturday

**Fig. 9:**
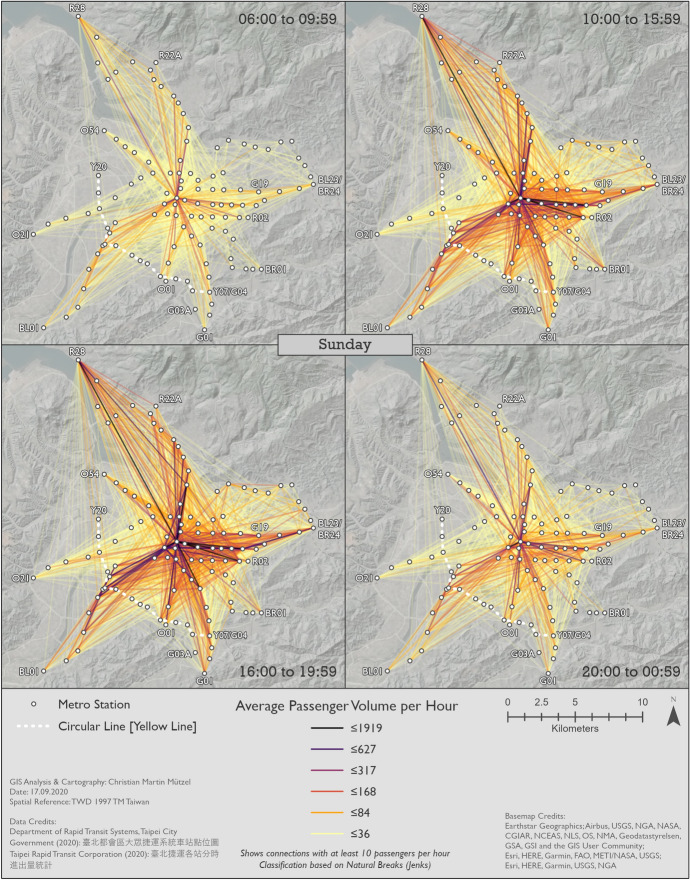
Station-to-station passenger flow in normal conditions on Sunday

Figures 7, 8 and 9 show the patterns in 2019 for Monday–Friday, Saturday, and Sunday, respectively. BL12/R10 Taipei Main Station can clearly be identified as the center on all maps. This station—like all other transfer stations—is only connected by two metro lines, but its central location and the transfer possibilities to high speed rail, regional train and Taoyuan Airport MRT help explain its importance. In general, center-to-center and center-to-periphery links have much higher passenger volumes than periphery-to-periphery links. A major exception, however, is R28 Tamsui station in the very north. Although this is the last station on the Tamsui–Xinyi Line [Red Line], it has strong interactions with many other stations. BL23/BR24 Taipei Nangang Exhibition Center station is not quite as remote as R28 Tamsui station, but also has strong interactions with other stations. This is probably due to the proximity of the commercial area Nangang Software Park and an exhibition center that hosts fairs. Furthermore, passengers can transfer to high speed rail and regional train here.

Although weekday morning and evening rush hours have very similar total passenger volumes, as already shown in Fig. 3, the morning trips are more equally spread across the network. This could be because in the morning people usually go directly to work or school, whereas in the afternoon they include other activities in a trip chain before returning home. Furthermore, the pattern during weekday rush hours is more equally spread across space than in all other time slices. While the volume of passenger flows on Saturday is higher than on Sunday, the spatial patterns remain largely the same. Even weekday noon and night show similarities to the respective times on weekdays, despite their differences in passenger volumes. Surprisingly, although the weekend has a lower number of total metro trips, there are more origin-destination links with extremely high passenger volumes than even during weekday rush hours.

#### Changes in metro usage under the impact of COVID-19

**Fig. 10:**
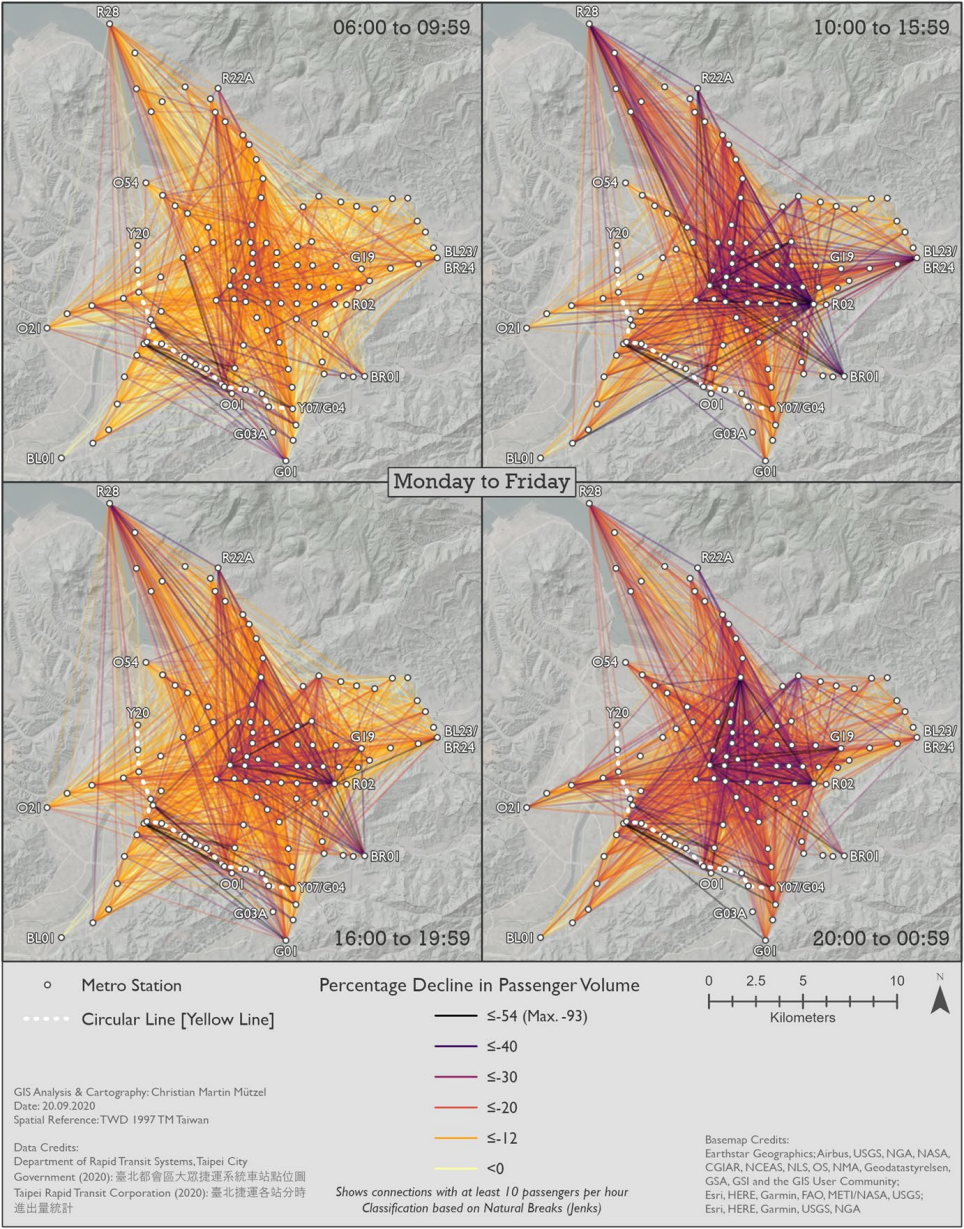
Station-to-station passenger flow under the impact of COVID-19 on Monday–Friday

**Fig. 11:**
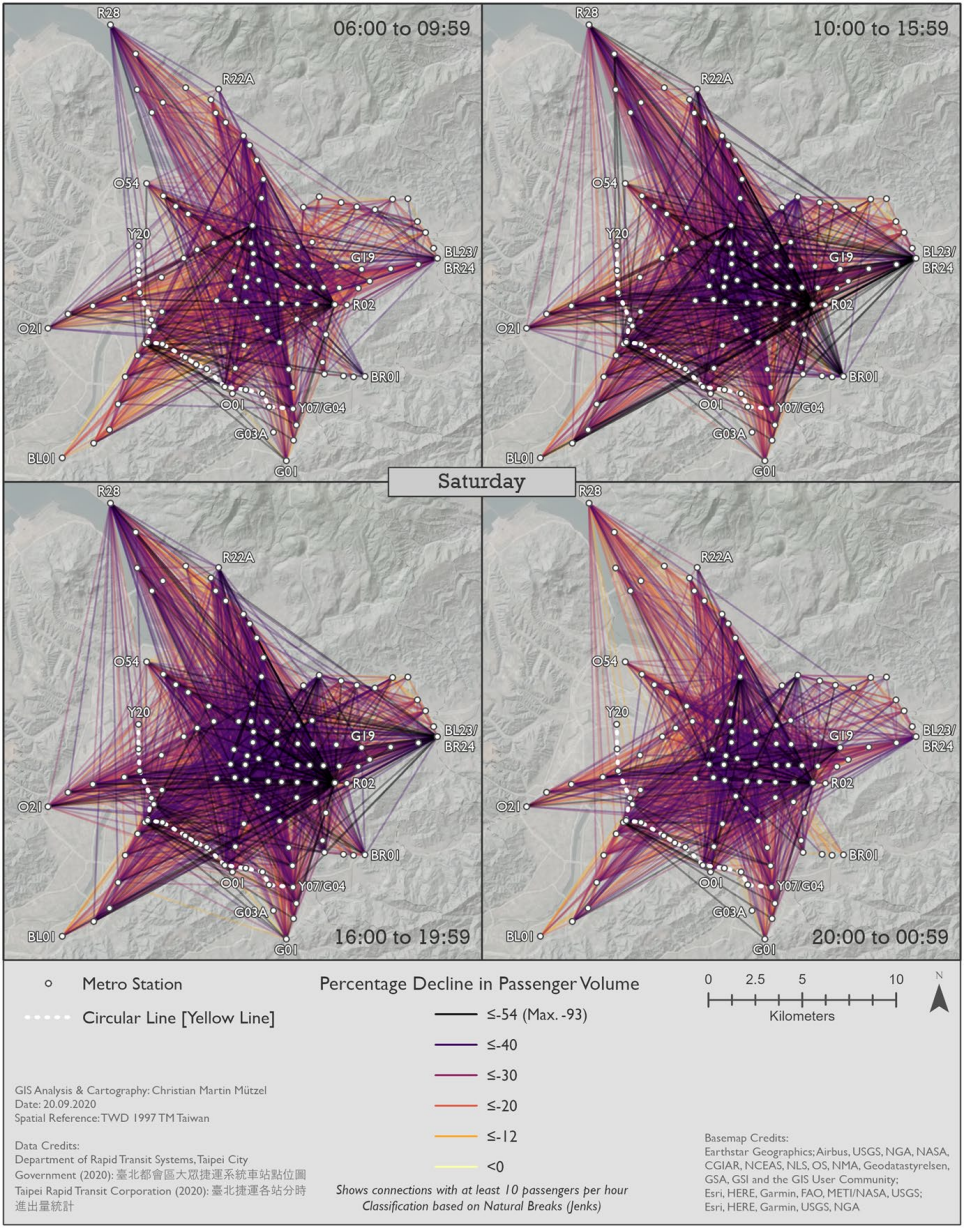
Station-to-station passenger flow under the impact of COVID-19 on Saturday

**Fig. 12:**
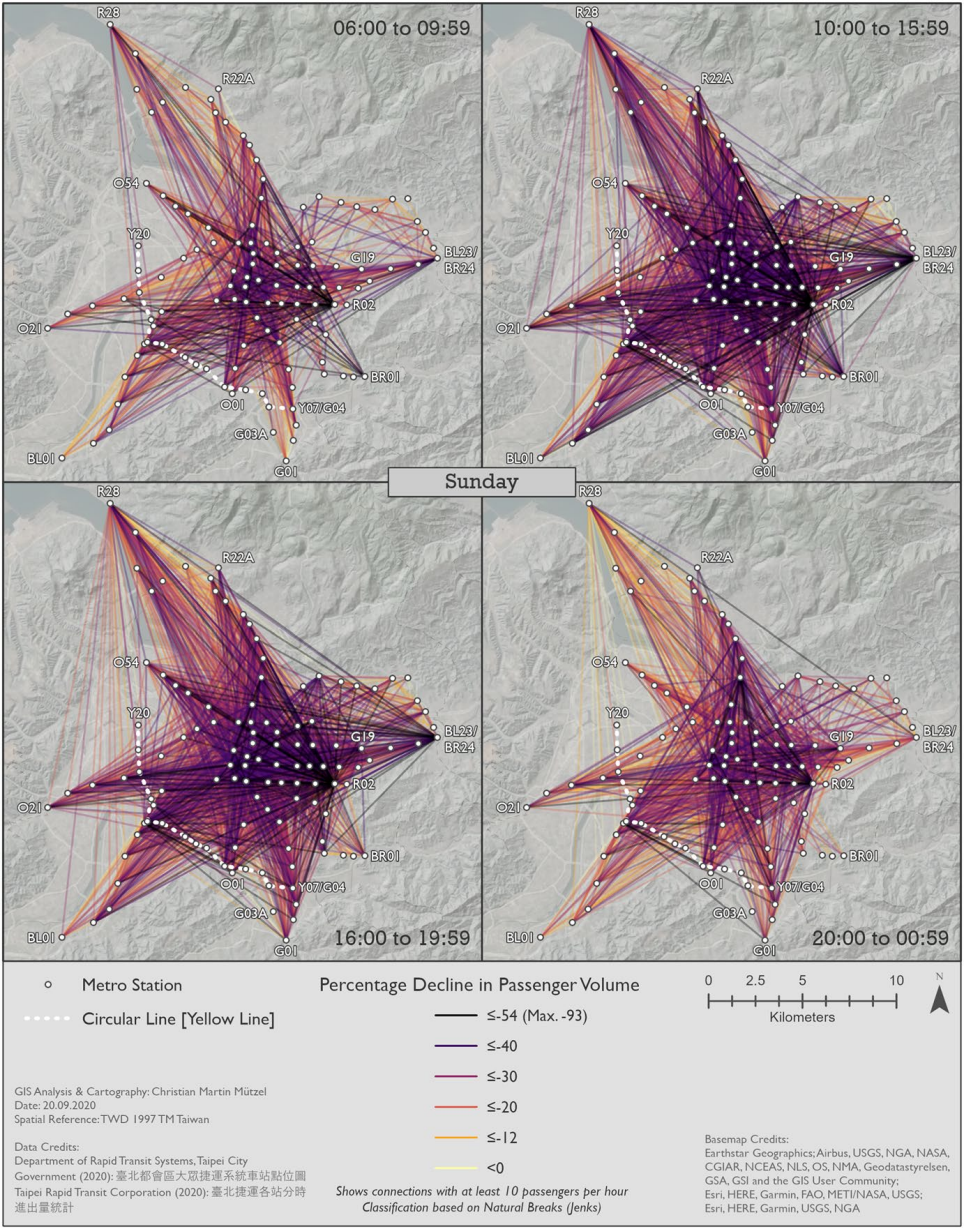
Station-to-station passenger flow under the impact of COVID-19 on Sunday

Figures 10, 11 and 12 show the percentage changes in metro usage from 2019 to 2020, i.e. under the impact of COVID-19 for Monday–Friday, Saturday, and Sunday, respectively. There are several links connecting the south-west and south with very high declines in passengers in all maps. Some of this strong decline is probably caused by the opening of the Circular Line [Yellow Line] rather than by COVID-19. Most of these origin-destination links include BL07 Banqiao station, next to which Y16 Banqiao station was built. The metro smart card data records these two as separate stations, thus explaining the high decline as passengers travelling on the links connecting south-west and south are now likely to use Y16 Banqiao station on the newly built Circular Line [Yellow Line] instead of BL07 Banqiao station on the Bannan Line [Blue Line].

In comparison to metro usage under normal conditions, the decline in metro passenger volume under the impact of COVID-19 is much more equally distributed across the network. It is more difficult to identify clear structures, and the patterns are both spatially and temporally very heterogeneous. Links which lost the largest proportion of passengers are not limited to central parts of Taipei City. Even many periphery-to-periphery links had many fewer passengers than before. Although relatively remote, links including BL23/BR24 Taipei Nangang Exhibition Center station emerge in several time slices (especially Monday noon, Saturday noon and evening, Sunday noon and evening) with an exceptional decrease in passengers in comparison to other areas. On weekdays at noon, in the evening and at night Xinyi CBD, Ximending CBD and R15 Jiantan station form a more or less pronounced triangle of metro trips with especially strong declines in passengers. One reason for R03 Taipei 101/World Trade Center station and BL23/BR24 Taipei Nangang Exhibition Center station experiencing such strong declines in passengers is probably that there are large exhibition centers near these stations where big fairs take place. Many fairs in the period analyzed were cancelled due to COVID-19. Furthermore, R28 Tamsui station and BR01 Taipei Zoo station often stand out: they are the last stations on their respective lines, thus relatively remote, but with high declines in passenger volumes. Despite the fact that patterns on weekends are highly diffuse, Xinyi CBD stands out in almost all time slices with an extremely high decline in passengers. In some time slices on weekends, Ximending CBD can also be identified as having many links that lost many more passengers than other areas. The overall patterns of Saturday and Sunday are similar, although there are differences in details.

In a temporal perspective, it is important to re-iterate a key result from Sect. 4.1; the decline in ridership is considerably stronger on weekend than workdays. On Mondays to Fridays the relative decline is weaker in the rush hours than in the 10–16 h and the 20–01 h time slices. It seems thus that work trips by public transit were not reduced as much as other, less mandatory trips.

## Conclusions

This study presented a detailed network-wide spatio-temporal analysis of Taipei Metro trip patterns using passively collected AFC smart card trip data. This included metro usage in normal conditions (January–March 2019) and changes in metro usage under the impact of COVID-19 (January–March 2020). Based on simple linear regression and line graphs, days of the week and times of the day with similar temporal passenger flow patterns were identified. Then, we created radial flow maps to visually examine station-to-station mobility patterns based on weekdays and daytimes. The results provide deep insights into the spatio-temporal characteristics of metro usage, which help public transit operators, transport planners and urban planners to better understand urban mobility as well as the impacts of COVID-19 on metro usage.

To the best of our knowledge, this study is the first to comprehensively map network-wide spatio-temporal metro usage at station-to-station level for a whole week divided into several time slices, while taking actual geography into account and clearly indicating magnitudes of passenger flow. Furthermore, this study is the first to comprehensively investigate spatio-temporal changes in metro usage under the impact of COVID-19 at the same level of detail. We showed that metro usage under normal conditions and the decline in metro usage under the impact of COVID-19 are both spatially and temporally highly heterogeneous. The applied mapping approach allows the direct visualization and analysis of these various heterogenous patterns.

Despite Taiwan's success in dealing with the COVID-19 pandemic, metro usage decreased strongly and seemed to stay at a lower level even after COVID-19 was fully controlled. This development poses a serious threat for public transit operators due to financial losses, and represents a risk for the transition to more sustainable and public transit-based cities. However, the data used do not permit to investigate whether the decline in passengers happened because people are going out less often, or because people changed to other modes of transportation—and if so, to which modes. If the former is the case then it is likely that public transit usage may recover and return to the pre-COVID-19 levels. Less traffic in general is not bad news for urban planners. Mode switches, on the other hand, especially to individual motorized modes, may have more lasting effects. This would be the ‘bad case’ scenario for public transit operators, urban planners and the environment, which could pose many challenges in the future. From the existing literature one may take that both factors are likely to be at play. On the one hand, working from home has become a 'new normal' to many in various countries, and this has reduced commuting (Follmer et al. 2020). On the other hand, mode switches to cycling and driving at the expense of transit use have been observed in various countries (Follmer et al. 2020; Molloy et al. 2021; Shibayama et al. 2021). Both observations may be lasting effects of COVID-19.

Since Taiwan has so far fully controlled the COVID-19 outbreak, public transit usage should continue to be closely monitored. Recent data for 2021 are available now and can be explored in future research. What happens in Taiwan—or other places that are in a similar situation—could indicate what public transit in cities around the world might face in the future after having more or less controlled COVID-19. The most important lesson to be learnt from COVID-19 is that such an outbreak can happen any time, and there is need to be prepared in the future. For public transport this may include to improve hygienic conditions in vehicles and at stations, more physical distancing between customers and, thus, more capacities, but also to develop new business models that may be needed if increased working from home relaxes the commitment to public transit in terms of owning season tickets. In addition, further studies should be carried out to investigate changes in mode choice and the level of mobility, and whether these changes are likely to be short-, medium-or long term.

## Footnotes

1 The Open Data is made available to the public under the Open Government Data License; users can make use of it provided they comply with the conditions and obligations of its terms. Open Government Data License: https://data.gov.tw/license.
